# Population Structure Analysis of Bull Genomes of European and Western Ancestry

**DOI:** 10.1038/srep40688

**Published:** 2017-01-13

**Authors:** Neo Christopher Chung, Joanna Szyda, Magdalena Frąszczak, The 1000 Bull Genomes Project, Hans Rudolf Fries, Mogens SandøLund, Bernt Guldbrandtsen, Didier Boichard, Paul Stothard, Roel Veerkamp, Michael Goddard, Curtis P. Van Tassell, Ben Hayes

**Affiliations:** 1 Biostatistics Group, Department of Genetics, Wroclaw University of Environmental and Life Sciences, Wroclaw, 51631, Poland; 2 Animal Breeding Department, Technical University Munich, Germany; 3 Department of Molecular Biology and Genetics, Aarhus University, Denmark; 4 French National Institute for Agricultural Research (INRA), France; 5 Department of Agricultural, Food and Nutritional Science, University of Alberta, Canada; 6 Department of Animal Breeding and Genetics, Wageningen University and Research Centre, Netherlands; 7 Department of Animal Genetics, the University of Melbourne, Australia; 8 United States Department of Agriculture, U.S.; 9 Centre for Animal Science, University of Queensland, Australia

## Abstract

Since domestication, population bottlenecks, breed formation, and selective breeding have radically shaped the genealogy and genetics of *Bos taurus*. In turn, characterization of population structure among diverse bull (males of *Bos taurus*) genomes enables detailed assessment of genetic resources and origins. By analyzing 432 unrelated bull genomes from 13 breeds and 16 countries, we demonstrate genetic diversity and structural complexity among the European/Western cattle population. Importantly, we relaxed a strong assumption of discrete or admixed population, by adapting latent variable models for individual-specific allele frequencies that directly capture a wide range of complex structure from genome-wide genotypes. As measured by magnitude of differentiation, selection pressure on SNPs within genes is substantially greater than that on intergenic regions. Additionally, broad regions of chromosome 6 harboring largest genetic differentiation suggest positive selection underlying population structure. We carried out gene set analysis using SNP annotations to identify enriched functional categories such as energy-related processes and multiple development stages. Our population structure analysis of bull genomes can support genetic management strategies that capture structural complexity and promote sustainable genetic breadth.

*Bos taurus* (cattle) has long experienced selection for high quality milk and meat production. To maintain and encourage genetic diversity, it is important to characterize the population structure of cattle. Inferring population structure and genetic differentiation play an increasingly important role in conservation efforts, genealogy, and selection programs. In this study, we have analyzed a large number of whole genome sequences of *Bos taurus* males (bulls) from 13 breeds, representing 16 countries, to characterize population structure and genetic diversity.

Recognizing the importance of cattle genome diversity in genome-wide association studies, genomic predictions, and optimal breeding, there have been substantial efforts to obtain genome-wide genotypes of multiple breeds in diverse geographical locations^1^,^2^,^3^. The 1000 Bull Genomes Consortium has successfully collaborated with institutions from more than 20 countries to collect 1577 whole genome sequences (as of version 5). Although the structural complexity of cattle has previously been studied based on array-based genome profiles or selected genetic markers, focusing on regions and breeds^4^,^5^,^6^,^7^,^8^,^9^, a population genomic study involving whole genome sequences related to European and Western ancestry has not been performed.

Moreover, most studies assumed discrete structure among representatives of a studied population, as defined by self-identified breeds. Recent studies using unsupervised classification, admixture models, and other techniques demonstrate greater structural complexity^1^,^2^,^8^, but direct estimation and utilization of population structure with relaxed assumptions have been challenging. Logistic factor analysis (LFA) uses recently developed probabilistic models of individual allele frequencies underlying genotypes that are appropriate for a wide range of population structures (e.g., discrete, continuous, or admixture)^10^. Building on principal component analysis (PCA), LFA provides a non-parametric estimation method tailored to large-scale genotype data. By modeling each single nucleotide polymorphism (SNP) by the population structure estimated by logistic factors (LFs), genetic differentiation can be directly tested and inferred.

Applying latent variable probabilistic models, we analyzed 432 unrelated *Bos taurus* genomes from 13 breeds and 16 countries, as part of the 1000 Bull Genomes Project^2^. This study provides detailed assessment of population structure among a diverse panel of whole genome sequences (~4.0 million SNPs per bull). We identified pervasive genetic differentiation as suggested by domestication and selection. Through incorporating gene set analyses with genomic features, evolutionary pressure on genetic variation is investigated. Additionally, we present an interactive visualization, which enables exploration of underlying population structure by LFs.

## Results

**Figure 1:**
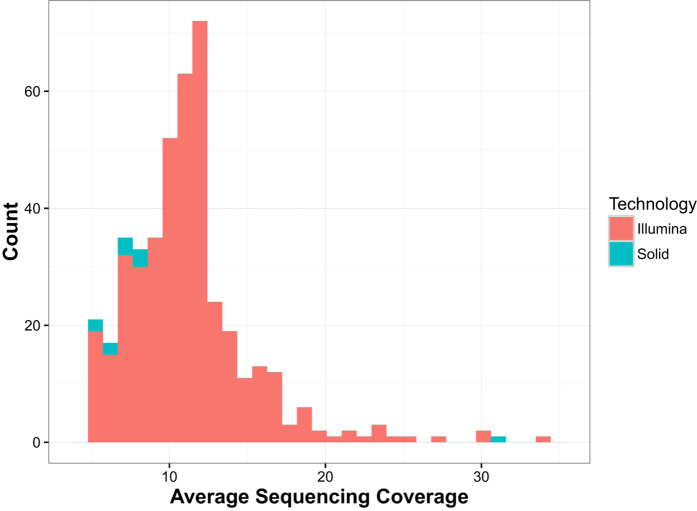
Average sequencing coverage of 432 bull samples. Samples with average sequencing coverage >5 are removed in a preprocessing step.

**Figure 2:**
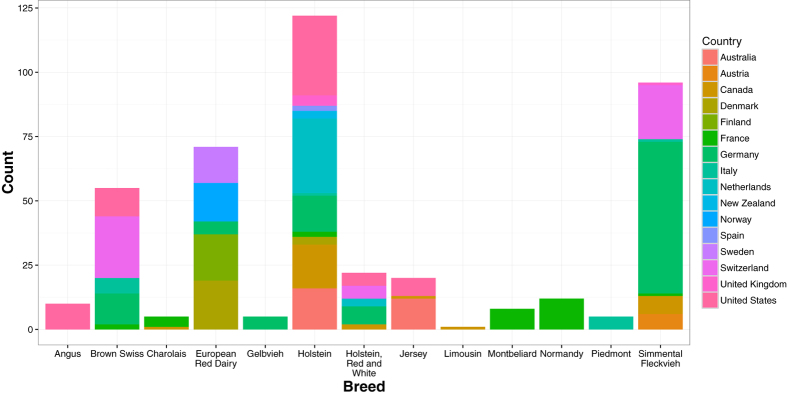
Bar plot of cattle breeds, with a number of samples colored by countries of origin.

In the 1000 Bull Genomes Project dataset, there were *n* = 432 unrelated *Bos taurus* samples with average sequencing coverage >5 (Fig. 1). These bulls represent 13 different European and Western breeds; namely, Angus, Brown Swiss, Charolais, Gelbvieh, Holstein, Jersey, Limousin, Montbeliard, Normandy, Piedmont, European Red Dairy, Holstein, Red & White, and Simmental/Fleckvieh. Defined by the official animal identification, our samples came from Australia, Austria, Canada, Denmark, Finland, France, Germany, Italy, Netherlands, New Zealand, Norway, Spain, Sweden, Switzerland, United Kingdom, and United States (Fig. 2). Among these genomes, there are *m* = 3,967,995 single nucleotide polymorphisms (SNPs) with no missing values and minor allele frequencies >0.05 (Supplementary Fig. 1).

**Figure 3:**
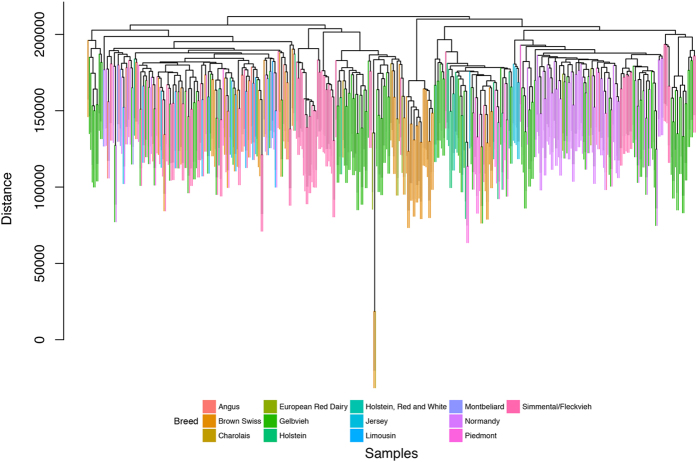
Hierarchical clustering of 432 bull genomes. Genome-wide SNPs are clustered using Manhattan distances and samples are colored by breeds.

To explore structural complexity, whole genome sequences of 432 selected samples were hierarchically clustered using Manhattan distances (Fig. 3, colored by 13 different breeds). Samples from the same breed do not necessarily appear together, although that does not imply whether breeds capture substantial and useful characteristics of bulls. Similarly, mutual k-nearest neighbour graphs (mkNNGs) were created by applying NetView^11^,^12^ for *k* = 6 and 12, where samples from different breeds are clustered together (Supplementary Fig. 2). Based on hierarchical clustering dendrogram and mkNNG clusters, it is evident that genetic structure may be more complex than breed codes.

**Figure 4:**
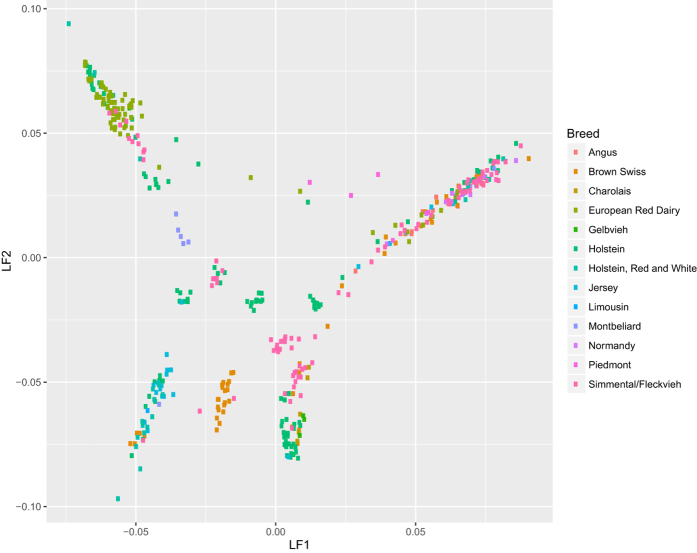
Scatterplots of the top two logistic factors (LFs). Data points corresponding to 432 bull genomes are colored by 13 breeds. Other scatterplots and interactive visualization are available at https://nnnn.shinyapps.io/bullstructure/.

The dimension of the population structure in logistic factor analysis (LFA) was set at *d* = 7, as estimated by the VSS algorithm and the scree plot of decreasing eigenvalues (Supplementary Fig. 3). The estimated logistic factors demonstrate the genetic continuum, reflecting shared origins of genetics and goals of breeding programs since domestication (Fig. 4). At the same time, the logistic factor 4 displays a clear distinction of Brown Swiss (from Switzerland, Germany, France, and Italy) and projection of logistic factors (LFs) allows straightforward visual identification of clusters (Supplementary Fig. 4). We enable interactive exploration of this population structure by creating an online app visualizing LFs according to user-specified parameters (https://nnnn.shinyapps.io/bullstructure/).

**Figure 5:**
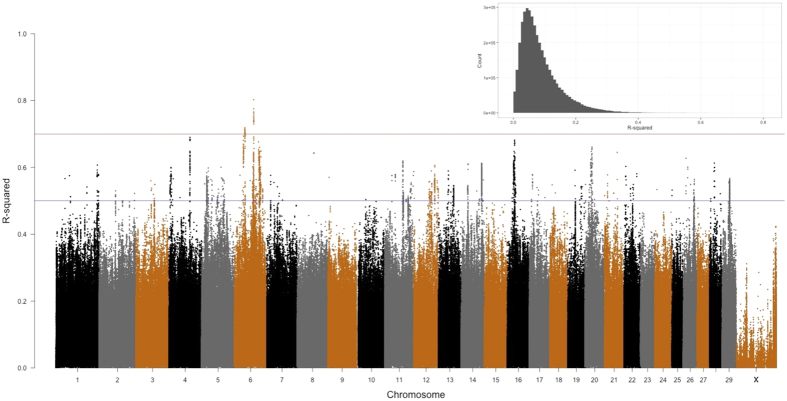
Genome-wide pseudo *R*^2^ measures with respect to logistic factors (LFs). The distribution is highly skewed towards 0, which leads to overplotting in a low range (see an insert for a genome-wide histogram). Overall, the median and mean are 0.070 and 0.087, respectively.

We discovered diverse and pervasive genetic differentiation with respect to the population structure of bulls. We found that the median and mean values of McFadden’s pseudo *R*^2^ (hereafter referred to as *R*^2^) are 0.070 and 0.087, respectively (Fig. 5). Chromosome 6 contained substantially more SNPs with high *R*^2^ than other chromosomes; it harbors 166 (39.0%) out of 426 SNPs with *R*^2^ > 0.6, as well as all 29 (100%) SNPs with *R*^2^ > 0.7. On the other hand, the X chromosome shows the least variation with respect to logistic factors, containing zero SNP with *R*^2^ > 0.5. The top 1000 genomic features that are associated with differentiated SNPs are shown in Supplementary Data 1.

Additionally, independent analyses were conducted to confirm robustness of our results. Particularly, we applied 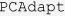 methodology on the same ~4.0 million SNPs, to identify SNPs under selection. In particular, after population structure is estimated by *k* = 6 PCs, communality statistics^13^ or Mahalanobis distances^14^ between each genomic variable and the top *k* PCs are used to detect local adaptation. Absolute correlation statistics between the top 6 LFs and the top 6 PCs were very high: 0.999, 0.894, 0.890, 0.994, 0.994, and 0.992 for each comparison between *i*^th^ LF and *i*^th^ PC for *i* = 1, …, 6. High concordance between the two methods can also be seen in a scatterplot of the top two PCs, compared to that of LFs (Supplementary Fig. 5). The Spearman correlation between *R*^2^ measures w.r.t. LFs and communality statistic w.r.t. PCs is 0.86, whereas that between *R*^2^ and Mahalanobis distances is 0.68. It may suggest that our method using McFadden’s pseudo *R*^2^ is more similar to communality statistic than Mahalanobis distances. Overall, the results from 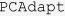 robustly support cattle population structure and genetic differentiation identified using LFA and *R*^2^.

Among SNPs with the highest *R*^2^ > 0.7, there exist two regions on chromosome 6; specifically 14 SNPs (13 within 50 kbp of known genomic features) positioned between 71101370 and 71600122 and 15 SNPs (11 within 50 kbp of known genomic features) positioned between 38482423 and 39140537. 83% of those most differentiated SNPs (20 out of 24 SNPs with known genomic features) are within or close to genes related to the selection sweep according to ref. 15. Among the first region, five SNPs fall within CHIC2 (ENSBTAG00000032660), while the closest features within 50 kbp also include GSX2 (ENSBTAG00000045812), U6 spliceosomal RNA (ENSBTAG00000042948), and novel pseudogene (ENSBTAG00000004082). U6 spliceosomal RNA (ENSBTAG00000042948) and novel pseudogene (ENSBTAG00000004082) are known to be associated with milk protein percentage^16^. In the second region, the exact overlaps occur in FAM184B (ENSBTAG00000005932), LCORL (ENSBTAG00000046561), and NCAPG (ENSBTAG00000021582). LCORL encodes a transcription factor whose human ortholog is involved in spermatogenesis, whereas NCAPG is crucial in mitosis and meiosis. Expecting much granular investigation of such genomic features, the list of 396,800 SNPs at the top 90 percentile (*R*^2^ > 0.174) is available as Supplementary Data 2.

To better understand evolutionary and biological processes, we conducted gene set analyses using genomic annotations of SNPs. Firstly, we found that SNPs located within known genomic features have about 1.8% higher *R*^2^ measures than intergenic SNPs without annotations (MWW p-value 9.85 × 10^−106^; Bonferroni corrected p-value 2.46 × 10^−106^). On the other hand, among intergenic SNPs, we found no significant correlation (p-value of 0.44) between SNP-feature distances and *R*^2^ measures (Supplementary Fig. 6). Secondly, among genic SNPs, *R*^2^ measures corresponding to SNPs within exons are slightly higher than those within introns by 0.27% with a MWW p-value 3.89 × 10^−29^ (Bonferroni corrected p-value 9.73 × 10^−28^). Start/stop codons and 3′/5′ UTR do not exhibit statistically significant difference from other genic SNPs. Lastly, we used 338 genes that are closest to SNPs with *R*^2^ > 0.5 in the 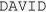 functional annotation tools. We found a total of 34 enriched annotation clusters, of which 11 clusters with enrichment scores >0.5 are shown in Table 1. Biological processes and functions related to calcium-binding domain (cluster 1 and 9) and iron containing hemeproteins related to ATP (cluster 3 and 6) exhibit strong enrichment, potentially reflecting causes of population structure. Notably, we observed functional clusters for sexual, respiratory, and embryonic development (cluster 5, 7, and 10, respectively).

## Discussion

*Bos taurus* has played a crucial role in ancient and modern societies alike by providing agricultural support and essential nutrients. Accurate characterization of its population structure helps conservation of genetic resources and optimal selection programs, ensuring a healthy and sustainable cattle population. In this process, we can better infer the genetic and functional variation that underlies the population structure. Using 432 samples from the 1000 Bull Genome Project, we provide a comprehensive sequencing-based assessment of population structure among cattle of European and Western ancestry.

Assumptions underlying population structure and its estimation methods have evolved to address growing genomics data in terms of complexity and scales^10^,^17^,^18^,^19^. Previous studies on genetic structure of cattle often model their samples as admixture of *k* ancestral populations. This critical choice of *k* depends on analytical solutions, such as log probability of data^18^, its rate of change^20^, or validation on independent test datasets (i.e., cross-validation)^21^. However, these methods may be sensitive to early divergence events or unable to capture hierarchical relationships^7^. Analysis of regional breeds often needs to include other published cattle genomes in order to estimate introgression or admixture^5^,^8^,^9^. This poses a significant challenge in population genomics.

We circumvent this challenge by using complementary methods that do not need to select *k* ancestral populations. Particularly, we utilize latent variable probabilistic models that can estimate a broad range of arbitrarily complex structure including admixture, continuous, and discrete population^10^. Some breeds are clearly distinguished by logistic factors (LFs), such as Brown Swiss by the fourth LF. However, LFs do not directly correspond to breeds or ancestral populations. To aid in comprehensively describing and exploring population structure from our analysis, we developed an interactive visualization app.

When modeling SNPs with logistic factors in generalized linear models, we found widespread genetic differentiation due to population structure. Despite making no assumption about structure, the majority of the most differentiated SNPs in our study have been identified as under selection sweep by previous studies. Chromosome 6, which harbors a large proportion of the highly differentiated SNPs, has been previously suggested to have been subjected to one or more selective sweeps^1^ and has also been associated with a number of milk and beef production traits^22^,^23^. Interestingly, given that the novel pseudogene (ENSBTAG00000004082), which has been known to be associated with calving performance^24^ and protein percentage^16^ is strongly associated with population structure, we suspect that it plays a crucial functional role in cattle genomes.

Our genome-wide study of differentiation suggests stronger evolutionary pressure on genic regions. Prolonged changes in environment, driven by domestication and development of cattle breeds, have likely caused genetic differentiation that focuses on functional regions of genomes^25^. Furthermore, enrichment analysis of genome annotations provides strong indications that functional groups related to energy production and development stages underlie the genes that are highly differentiated with respect to population structure.

This study paves a way to further our understanding of population structure among modern European and Western cattle breeds. Identification of genetic differentiation with respect to population structure may inform conservation efforts to preserve heritage breeds and maintain genetic diversity. Methodologically, our sequencing-based analysis of population structure represents non-parametric approaches that can identify genetic differentiation and complexity without strong assumption on structure in population genomics.

## Methods

### Bull Genomes

The 1000 Bull Genomes Project has collaborated to gather whole-genome sequences of breeds from Australia, Austria, Canada, Denmark, Finland, France, Germany, Italy, Netherlands, New Zealand, Norway, Spain, Sweden, Switzerland, and United Kingdom. Its initial efforts have vastly expanded known single nucleotide polymorphisms (SNPs) and copy number variations (CNVs) in *Bos taurus*^2^. Currently, it covers 1577 bull samples as of version 5 released in 2015, among which 1507 and 70 bull genomes were sequenced with Illumina/Solexa and ABI SOLiD technology, respectively. For analysis of population structure, we selected unrelated bulls with average sequencing coverage greater than 5. Among sibs only one representative was selected randomly. SNP genotypes were identified prior to our study based on whole genome sequence data of bulls, using a multi-sample variant calling procedure. Polymorphisms with minor allele frequencies below 0.05 were removed from analyses. For processing whole-genome sequences, we used 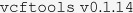
26, 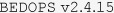
27, and 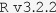
28.

### Statistical Analysis

To initially explore the genome-wide SNP data, we employ hierarchical clustering which enables straightforward visualization of relationships among samples. In particular, similarities/dissimilarities among 10% of 4.0 million SNPs are represented by Manhattan distances,


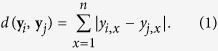


To hierarchically cluster samples, UPGMA (Unweighted Pair Group Method with Arithmetic Mean) is applied to Manhattan distances^29^. When visualizing a resulting dendrogram, nodes are colored by breed codes. Alternatively, we applied 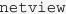 to create mutual k-nearest neighbour graphs (mkNNGs) based on the same set of SNPs^11^,^12^. Unlike hierarchical clustering, mKNNGs assign discrete memberships, which are visualized in a force-directed graph (as implemented in 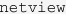).

To infer population structure directly from a genome-wide genotype matrix, we consider a probabilistic model of individual allele frequencies. In particular, by using logistic factor analysis^10^ that captures systematic variation of individual-specific allele frequencies arising from discrete or continuous sub-population, spatial variation, admixture, and other structures, we relax statistical assumptions imposed on bulls by its official breed and country code defined in the animal registration ID. While the statistical models and algorithms are extensively described elsewhere^10^, we provide a brief overview of this approach here.

Consider a genotype matrix **Y** with *m* SNPs and *n* bulls. For each *y*_*ij*_, an individual-specific allele frequency for *i*^th^ SNP and *j*^th^ bull is *f*_*ij*_ ∈ [0, 1]. This collection of parameters (a *m* × *n* **F** matrix) is transformed into real numbers via the logit function, which allows computation of the underlying latent structure. Overall, the statistical model considered is


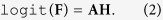


Then, the population structure is captured by *d* logistic factors (LFs) **H** which can be estimated by applying principal component analysis (PCA) to 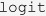 (**F**). Note that **A** is a matrix of coefficients in a logistic regression. The dimensions of logistic factors are estimated by comparing the observed correlation matrix to a series of hypothesized structures derived from selected variables of large loadings^30^. In the Very Simple Structure (VSS) algorithm, we considered *d* = 1, …, 100, while applying principal component analysis on the mean-centered genotypes (R package 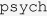). Eigenvalues of *m*^−1^ **Y**^*T*^ **Y** and percent variance explained by each component are visually inspected for the inflection point (e.g., elbow). For robustness analysis to confirm genetic differentiation, we alternatively used cross-validation approximations to choose *d*^31^.

To approximate how much of the variation in genotypes is explained by the population structure, we calculate McFadden’s pseudo *R*^2^ that is appropriate for a logistic regression^32^. For *i*^th^ SNP,


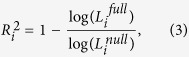


where 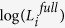 and 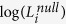 are maximum log-likelihoods of the full and null models, respectively. As this study only considers McFadden’s pseudo *R*^2^ in logistic regressions, we will henceforth refer to it as *R*^2^ when clear in context. Significance analysis with respect to logistic factors (or principal components) are done with a resampling-based jackstraw method^33^.

Additionally, we performed genome-wide scan for selection in the panel of SNP data using 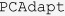
13,14,34. Generally, 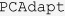 uses Mahalanobis distances and communality statistics between SNPs and the first *k* principal components (PCs), with appropriate normalization specific to each measure. Selection is detected when SNPs (or other genetic markers) are substantially explained by the first *k* PCs^13^,^34^. To evaluate concordance of results from 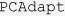 and LFA, we compute Spearman correlation between Mahalanobis/communality statistics using PCs and McFadden’s pseudo *R*^2^ measures using LFs.

### Annotation and Enrichment

For genome annotation, we used the latest *Bos taurus* reference genome from the Center for Bioinformatics and Computational Biology, University of Maryland (downloaded from the NCBI server ftp://ftp.ncbi.nlm.nih.gov/, version UMD3.1.83).

When testing whether the distribution of McFadden’s pseudo *R*^2^ measures are significantly different according to feature types, we used the Mann-Whitney-Wilcoxon (MWW) test^35^. With a large sample size, a Normal approximation is used to compute MWW p-values. In particular, we investigated whether SNPs falling within genes may have a higher McFadden’s pseudo *R*^2^ than those in intergenic regions. Among SNPs with known feature assignments, MWW tests were used to infer if a particular feature type is associated with significantly higher *R*^2^ measures. Bonferroni correction is applied on a set of four MWW tests to adjust for multiple hypotheses testing^36^,^37^.

Lastly, because some of SNPs are in intergenic regions with no known annotations, we utilized the closest features function from 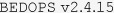
27. Among the top genes with McFadden’s pseudo *R*^2^ > 0.5, we apply 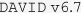 considering GO, KEGG pathways, InterPro, SwissProt Protein Information Resource, and other databases to identify enrichment of biological processes and functional pathways^38^. For intergenic SNPs, we searched the reference genome for the closest genes, which were used in 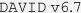. When clustering functional annotations, we set “Classification Stringency” to high.

## Additional Information

**How to cite this article**: Chung, N. C. *et al*. Population Structure Analysis of Bull Genomes of European and Western Ancestry. *Sci. Rep.* **7**, 40688; doi: 10.1038/srep40688 (2017).

**Publisher's note:** Springer Nature remains neutral with regard to jurisdictional claims in published maps and institutional affiliations.

## Supplementary Material

Supplementary Information

## Figures and Tables

**Table 1:** Enriched functional clusters, for genes associated with *R* ^2^ > 0.5.

Category	Term	Count	%	P Value
**Cluster 1**	**Enrichment Score: 1.405**	**Calcium-binding domain**		
INTERPRO	IPR018247:EF-HAND 1	6	2.098	0.035
INTERPRO	IPR018249:EF-HAND 2	6	2.098	0.038
INTERPRO	IPR011992:EF-Hand type	6	2.098	0.045
**Cluster 2**	**Enrichment Score: 1.372**	**Cysteine-type activity**		
GOTERM_MF_FAT	GO:0004198 ~ calcium-dependent cysteine-type endopeptidase activity	3	1.049	0.011
GOTERM_MF_FAT	GO:0008234 ~ cysteine-type peptidase activity	4	1.399	0.066
GOTERM_MF_FAT	GO:0004197 ~ cysteine-type endopeptidase activity	3	1.049	0.106
**Cluster 3**	**Enrichment Score: 0.897**	**Cytochrome**		
PIR_SUPERFAMILY	PIRSF000045:cytochrome P450 CYP2D6	3	1.049	0.013
INTERPRO	IPR002401:Cytochrome P450, E-class, group I	3	1.049	0.068
INTERPRO	IPR017973:Cytochrome P450, C-terminal region	3	1.049	0.080
INTERPRO	IPR017972:Cytochrome P450, conserved site	3	1.049	0.084
SP_PIR_KEYWORDS	heme	4	1.399	0.091
INTERPRO	IPR001128:Cytochrome P450	3	1.049	0.107
SP_PIR_KEYWORDS	Monooxygenase	3	1.049	0.124
COG_ONTOLOGY	Secondary metabolites biosynthesis, transport, and catabolism	3	1.049	0.148
GOTERM_MF_FAT	GO:0020037 ~ heme binding	4	1.399	0.159
GOTERM_MF_FAT	GO:0046906 ~ tetrapyrrole binding	4	1.399	0.176
GOTERM_MF_FAT	GO:0009055 ~ electron carrier activity	4	1.399	0.301
SP_PIR_KEYWORDS	iron	4	1.399	0.399
GOTERM_MF_FAT	GO:0005506 ~ iron ion binding	4	1.399	0.614
**Cluster 4**	**Enrichment Score: 0.860**	**Signaling**		
UP_SEQ_FEATURE	signal peptide	19	6.643	0.048
SP_PIR_KEYWORDS	signal	19	6.643	0.111
SP_PIR_KEYWORDS	glycoprotein	16	5.594	0.492
**Cluster 5**	**Enrichment Score: 0.833**	**Sexual development**		
GOTERM_BP_FAT	GO:0045137 ~ development of primary sexual characteristics	3	1.049	0.117
GOTERM_BP_FAT	GO:0003006 ~ reproductive developmental process	4	1.399	0.151
GOTERM_BP_FAT	GO:0007548 ~ sex differentiation	3	1.049	0.180
**Cluster 6**	**Enrichment Score: 0.760**	**Ion binding**		
GOTERM_MF_FAT	GO:0043167 ~ ion binding	40	13.986	0.130
GOTERM_MF_FAT	GO:0046872 ~ metal ion binding	38	13.287	0.190
GOTERM_MF_FAT	GO:0043169 ~ cation binding	38	13.287	0.213
**Cluster 7**	**Enrichment Score: 0.725**	**Respiratory development**		
GOTERM_BP_FAT	GO:0030324 ~ lung development	3	1.049	0.145
GOTERM_BP_FAT	GO:0030323 ~ respiratory tube development	3	1.049	0.145
GOTERM_BP_FAT	GO:0060541 ~ respiratory system development	3	1.049	0.150
GOTERM_BP_FAT	GO:0035295 ~ tube development	3	1.049	0.400
**Cluster 8**	**Enrichment Score: 0.723**	**Protease activity**		
GOTERM_MF_FAT	GO:0004175 ~ endopeptidase activity	8	2.797	0.129
GOTERM_MF_FAT	GO:0070011 ~ peptidase activity, acting on L-amino acid peptides	9	3.147	0.190
GOTERM_MF_FAT	GO:0008233 ~ peptidase activity	9	3.147	0.215
GOTERM_BP_FAT	GO:0006508 ~ proteolysis	12	4.196	0.242
**Cluster 9**	**Enrichment Score: 0.703**	**Calcium-binding domain**		
UP_SEQ_FEATURE	calcium-binding region:2	3	1.049	0.126
INTERPRO	IPR002048:Calcium-binding EF-hand	4	1.399	0.148
UP_SEQ_FEATURE	calcium-binding region:1	3	1.049	0.157
SMART	SM00054:EFh	4	1.399	0.187
UP_SEQ_FEATURE	domain:EF-hand 1	3	1.049	0.258
UP_SEQ_FEATURE	domain:EF-hand 2	3	1.049	0.258
INTERPRO	IPR018248:EF hand	3	1.049	0.333
**Cluster 10**	**Enrichment Score: 0.668**	**Embryonic development**		
GOTERM_BP_FAT	GO:0001824 ~ blastocyst development	3	1.049	0.082
GOTERM_BP_FAT	GO:0001701 ~ in utero embryonic development	4	1.399	0.165
GOTERM_BP_FAT	GO:0043009 ~ chordate embryonic development	4	1.399	0.397
GOTERM_BP_FAT	GO:0009792 ~ embryonic development ending in birth or egg hatching	4	1.399	0.400
**Cluster 11**	**Enrichment Score: 0.565**	**Cardiomyopathy**		
KEGG_PATHWAY	bta05412:Arrhythmogenic right ventricular cardiomyopathy (ARVC)	3	1.049	0.240
KEGG_PATHWAY	bta05410:Hypertrophic cardiomyopathy (HCM)	3	1.049	0.277
KEGG_PATHWAY	bta05414:Dilated cardiomyopathy	3	1.049	0.304
**Cluster 12**	**Enrichment Score: 0.519**	**Phosphorylation**		
GOTERM_MF_FAT	GO:0004672 ~ protein kinase activity	9	3.147	0.213
GOTERM_BP_FAT	GO:0006468 ~ protein amino acid phosphorylation	9	3.147	0.291
GOTERM_BP_FAT	GO:0016310 ~ phosphorylation	9	3.147	0.447
