# Supplementary material for: Population Structure Analysis of Bull Genomes of European and Western Ancestry
Source: Sci Rep. 2017 Jan 13;7:40688. doi: 10.1038/srep40688 (PMC5234001; doi:10.1038/srep40688)
Supplement: Supplementary Information [file srep40688-s1.pdf]

Supplementary Information: Population Structure Analysis of Bull Genomes of European and Western Ancestry

Neo Christopher Chung[1,*], Joanna Szyda[1], Magdalena Frąszczak[1], and the 1000 Bull Genomes Project[+]

[1]Biostatistics Group, Department of Genetics, Wroclaw University of Environmental and Life Sciences, Wroclaw, 51631, Poland
[*]nchchung@gmail.com

[+]The membership of the 1000 Bull Genomes Project are: Hans Rudolf Fries[2], Mogens SandøLund[3], Bernt Guldbrandtsen[3], Didier Boichard[4], Paul Stothard[5], Roel Veerkamp[6], Michael Goddard[7], Curtis P Van Tassell[8], and Ben Hayes[9].

[2]Animal Breeding Department, Technical University Munich
[3]Department of Molecular Biology and Genetics, Aarhus University
[4]French National Institute for Agricultural Research (INRA)
[5]Department of Agricultural, Food and Nutritional Science, University of Alberta
[6]Department of Animal Breeding and Genetics, Wageningen University and Research Centre
[7]Department of Animal Genetics, the University of Melbourne
[8]United States Department of Agriculture
[9]Centre for Animal Science, University of Queensland

1 Supplementary Figures

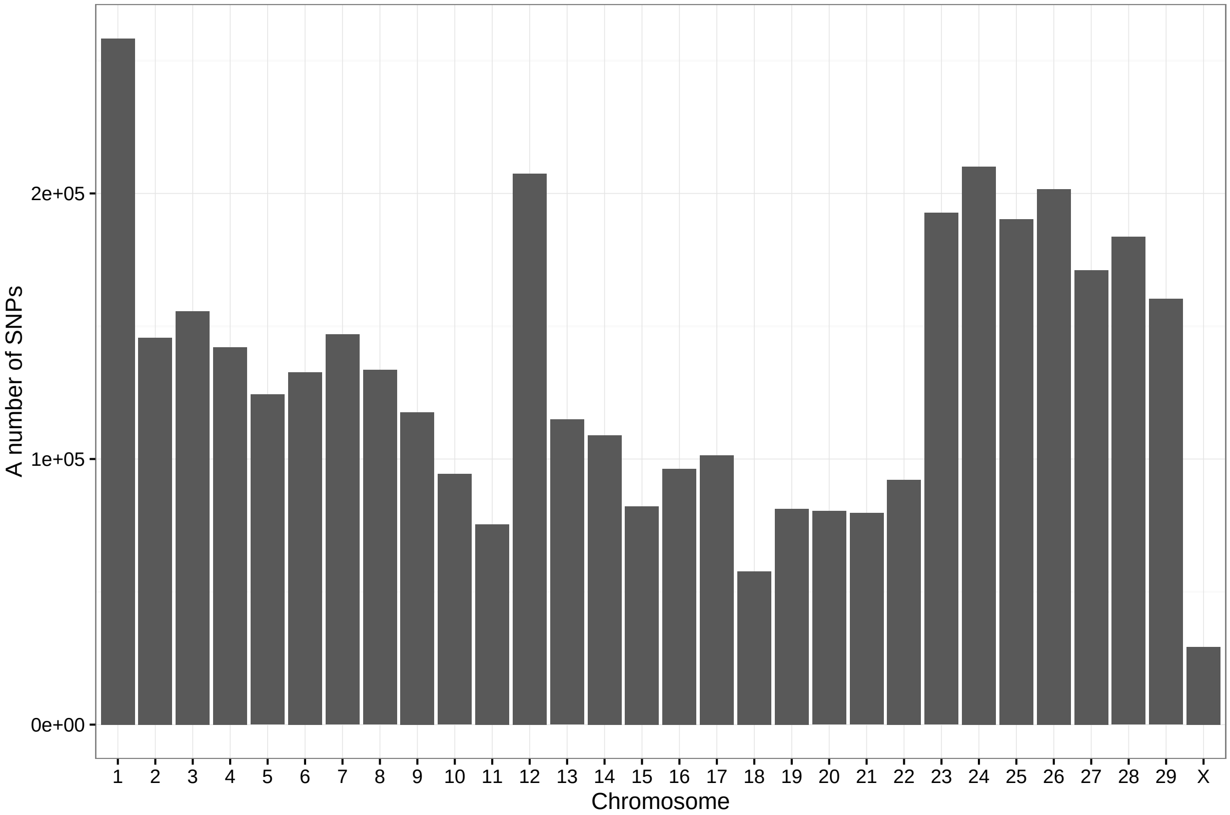

Supplementary Figure 1: Bar plot of numbers of SNPs on *Bos taurus* chromosomes analyzed in the study. We had chosen complete SNPs with minor allele frequencies > 0.05.

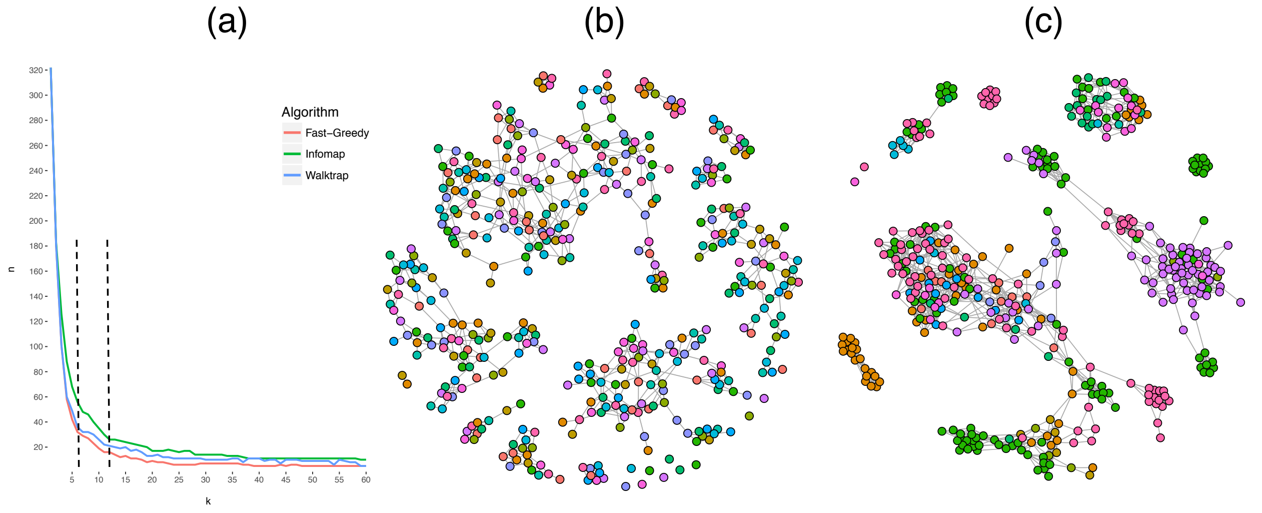

Supplementary Figure 2: Mutual k-nearest neighbor graphs (mkNNG) of bull genomes. Alternative to hierarchal clustering, mkNNG of `netview` software was applied on 10% of 4.0 million SNPs. *(a)* Since `netview` depends on a user-choice of k, three different community detection algorithms are used to assess an elbow. Potential elbows are marked by two dashed lines. *(b)* mkNNG of 432 bull samples using $k = 6$. *(c)* mkNNG of 432 bull samples using $k = 12$. Samples from the same cattle breed are colored together.

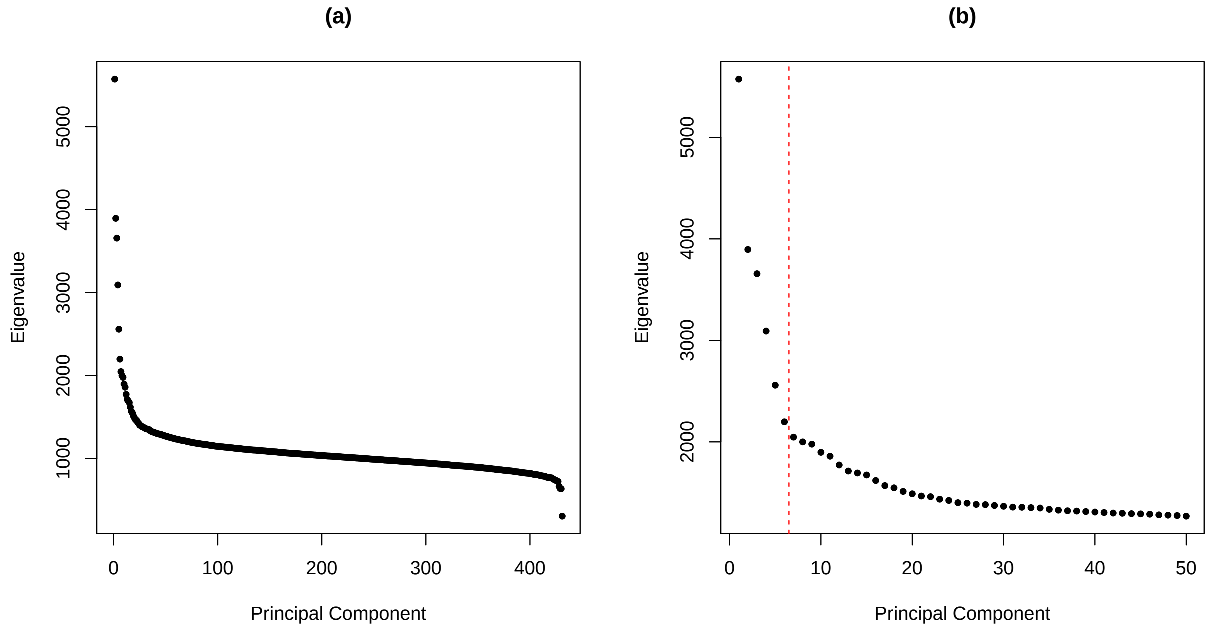

Supplementary Figure 3: Scree plot of the genome-wide SNP matrix. Eigenvalues of the mean-centered genome-wide SNP matrix are plotted in a descending order, with *(a)* showing all eigenvalues and *(b)* the top 50 eigenvalues. The red dashed line is drawn to indicate a dimension determined by the VSS algorithm.

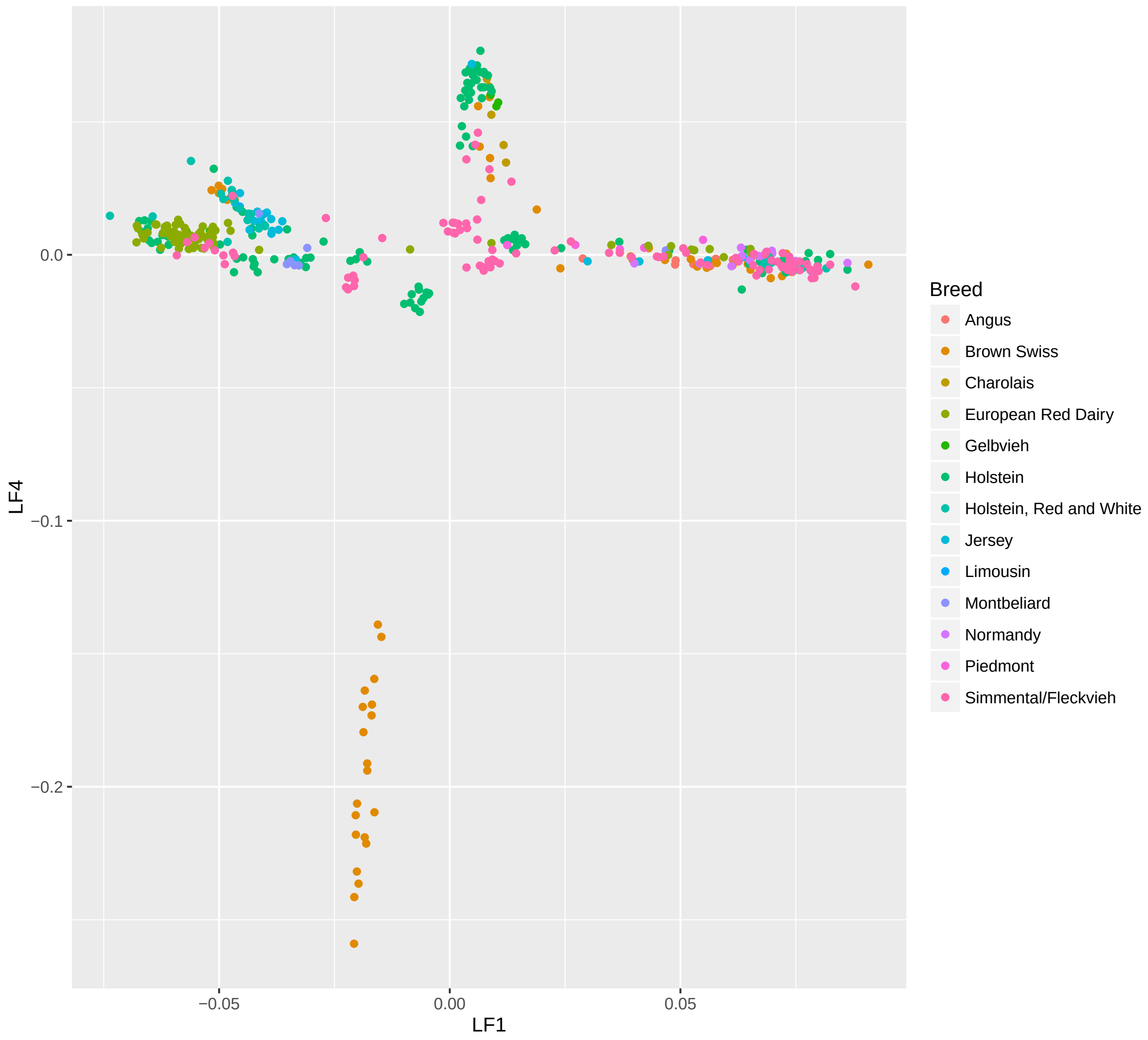

Supplementary Figure 4: Scatterplot of the 1st LF and 4th LF, clearly separating Brown Swiss from other breeds. Data points corresponding to 432 bull genomes are colored by 13 breeds.

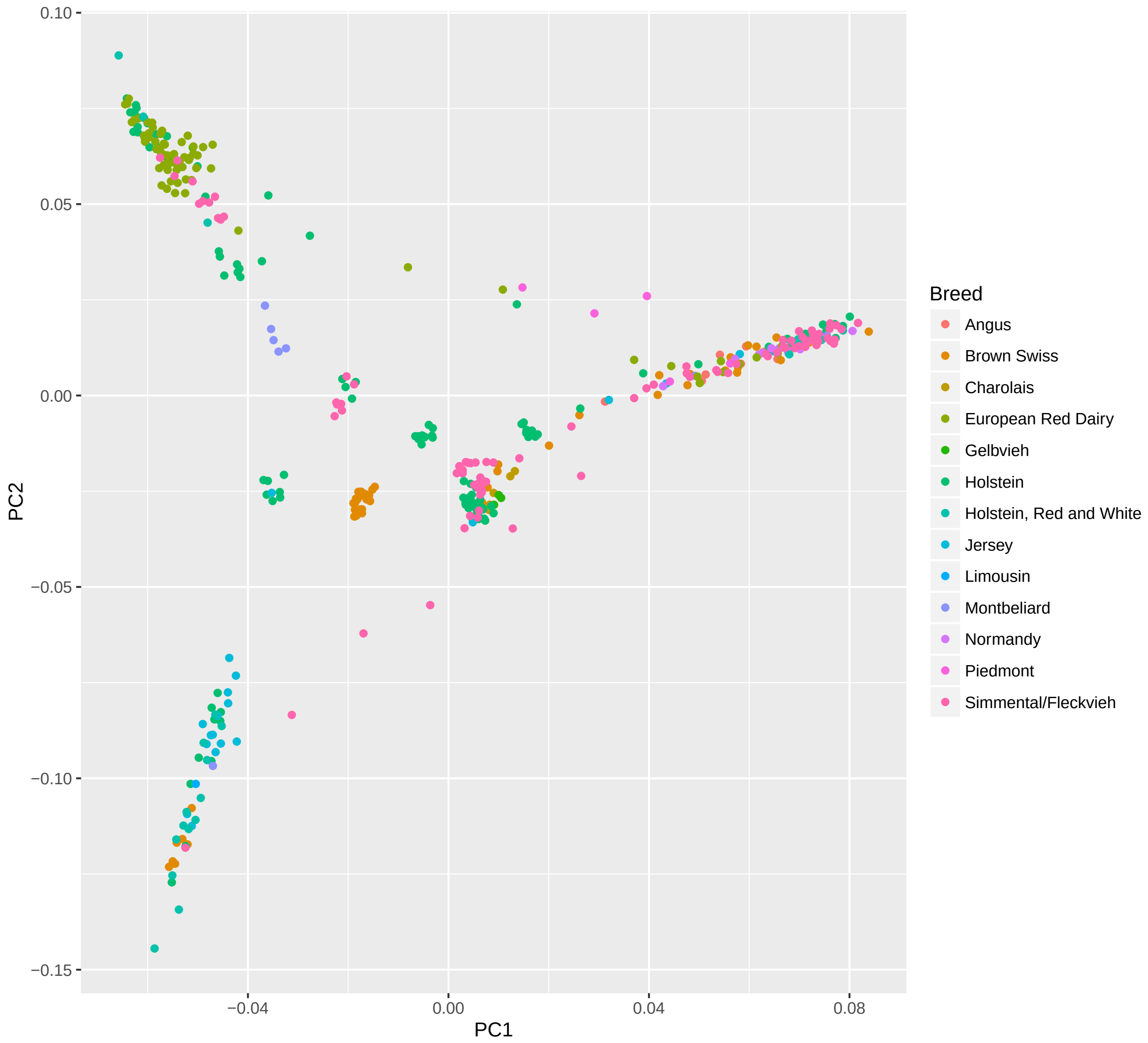

Supplementary Figure 5: Scatterplot of the top two principal components (PCs) from applying PCAdapt to SNP data. Data points corresponding to 432 bull genomes are colored by 13 breeds.

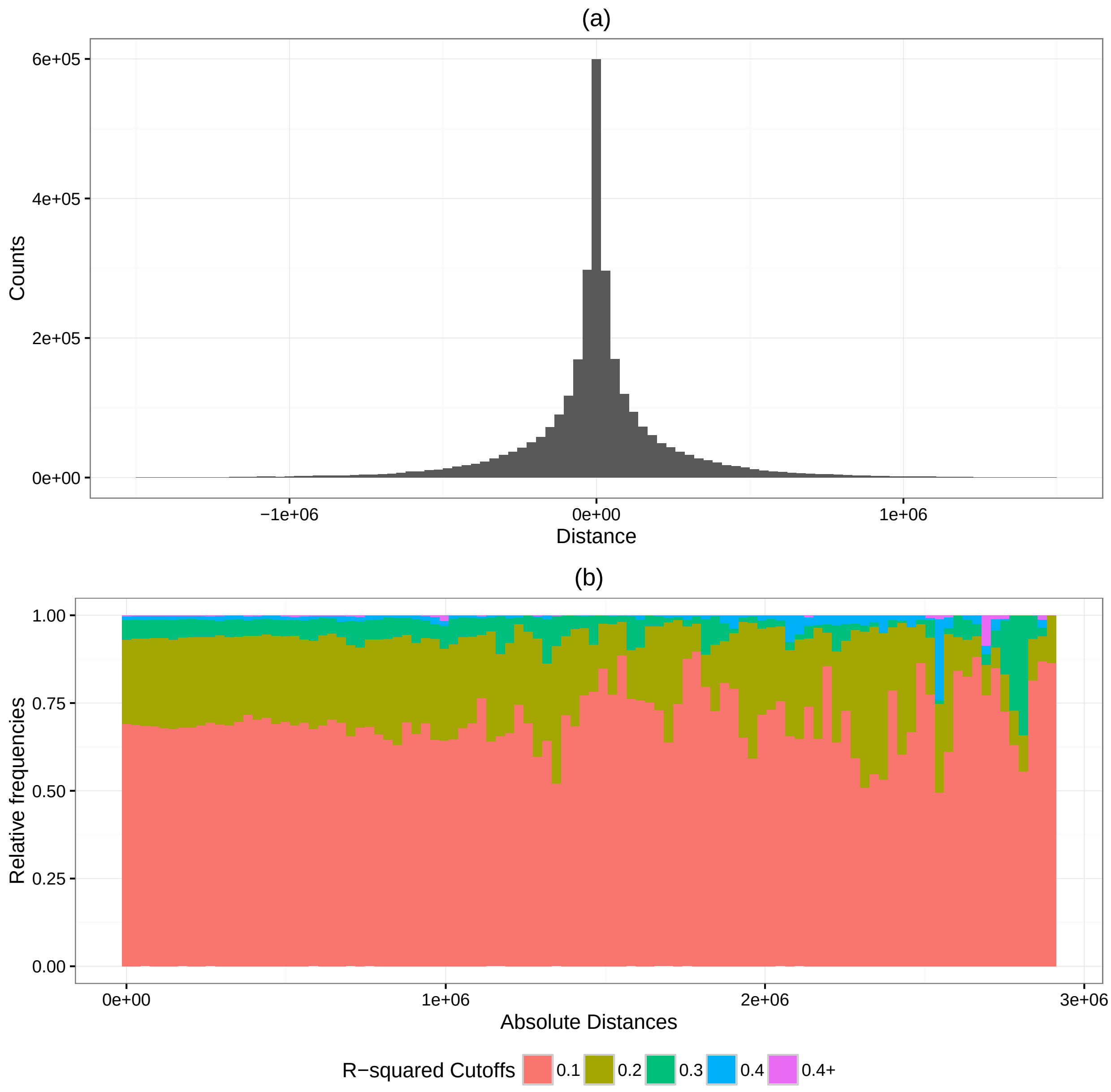

Supplementary Figure 6: *(a)* A distribution of distances between SNPs and their closest genomic feature. Outside of a shown x-axis range $(-1500000, 1500000)$, there are 13359 SNPs (.3% of total SNPs). *(b)* Relationship between absolute distances and McFadden's pseudo R^2. "R-squared Cutoffs" are upper thresholds of R^2 measures, such that a red label (R-squared Cutoffs of 0.1) refers to an amount of SNPs with $0 < R^2 < 0.1$. Overall, a Pearson correlation between distance and R^2 measures is $r = -0.0004$ (p-value of $= 0.44$).

2 Supplementary Data

`BullGenomesStructure_Top1000Features.csv (84,753 bytes)`
Supplementary Data 1: The top 1000 genomic features that are associated with the highly differentiated SNPs. When a given genomic feature is associated with multiple SNPs, the McFadden's pseudo highest R^2 corresponding to the most differentiated SNP is reported.

`BullGenomesStructure_SNPs.csv (19,860,346 bytes)`
Supplementary Data 2: The list of $396,800$ SNPs at the top 90 percentile of $R^2 > .174$.
